# The Water Suitcase of Migrants: Assessing Virtual Water Fluxes Associated to Human Migration

**DOI:** 10.1371/journal.pone.0153982

**Published:** 2016-04-28

**Authors:** Rodolfo Metulini, Stefania Tamea, Francesco Laio, Massimo Riccaboni

**Affiliations:** 1 Scuola Superiore S.Anna - Institute of Economics, Piazza Martiri della Libertà, 33, 56127 Pisa, Italy; 2 IMT Institute for Advanced Studies Lucca - Laboratory of Innovation Management and Economics, Piazza San Francesco 19, 55100 Lucca, Italy; 3 Politecnico di Torino - Dept. of Environment, Land and Infrastructure Engineering, Corso Duca degli Abruzzi 24, 10129 Torino, Italy; 4 KU Leuven - Department of Management, Stategy and Innovation, Naamsestraat 69, 3000 Leuven, Belgium; National Institute of Health, ITALY

## Abstract

Disentangling the relations between human migrations and water resources is relevant for food security and trade policy in water-scarce countries. It is commonly believed that human migrations are beneficial to the water endowments of origin countries for reducing the pressure on local resources. We show here that such belief is over-simplistic. We reframe the problem by considering the international food trade and the corresponding virtual water fluxes, which quantify the water used for the production of traded agricultural commodities. By means of robust analytical tools, we show that migrants strengthen the commercial links between countries, triggering trade fluxes caused by food consumption habits persisting after migration. Thus migrants significantly increase the virtual water fluxes and the use of water in the countries of origin. The flux ascribable to each migrant, i.e. the “water suitcase”, is found to have increased from 321 m^3^/y in 1990 to 1367 m^3^/y in 2010. A comparison with the water footprint of individuals shows that where the water suitcase exceeds the water footprint of inhabitants, migrations turn out to be detrimental to the water endowments of origin countries, challenging the common perception that migrations tend to relieve the pressure on the local (water) resources of origin countries.

## Introduction

Human migrations, dislocating food demand, impact the international trade of agricultural commodities and the food security of countries. The nexus between food, trade and human migration has been highlighted more and more frequently in the public debate. For example the worsening of the food security situation in the Near East is not only caused by structural constraints to food production and by the increasing dependence on food imports, but by conflicts, the flow of refugees and migration [1]. The network and dynamics of human migration have been recently and extensively studied [2, 3] and the relation between migration and trade has also been investigated [4, 5]. However, despite the attention received by the trade-migration relationship, the nexus between human migration, food trade and water resources for food production has not been addressed yet.

The production of food requires large amounts of water: 85% of freshwater consumed by human societies is ascribable to agriculture [6]. When commodities are traded, the water used for their production is virtually displaced by them, thus leading to flows of *virtual water* which are estimated to total 2320 m^3^/y, i.e, nearly 25% of the water globally consumed by humanity [7]. These numbers shed light on the pressure of mankind on global freshwater resources and justify the increasing interest towards this form of environmental impact, usually known as *water footprint*, which quantifies humans’ appropriation of freshwater resources.

The virtual water concept has been originally proposed by Allan [8] basing on the observation that food import of water scarce countries implies an import of the water “embedded” in the traded commodities. Reimer [9] gives economic foundations to the virtual water concept through the international trade theory of Heckscher-Ohlin-Vanek, according to which commodity trade can be seen as an implicit exchange of the factors of production “embedded” in the commodities in line with the interpretation of the factor content of trade [10].

The virtual water trade (VWT) and the corresponding network have been investigated in a number of recent studies [11–17]. It has been highlighted that VWT can contribute to food security by allowing water-scarce countries to benefit from water resources available elsewhere and to meet the food (and associated water) requirements of a growing population [18, 19]. VWT also determines water savings at the global scale, when it provides goods with lower water footprint than they would have if produced locally (e.g., [20]); however, it determines an externalization of resources, an increase in country interdependency and, possibly, a reduced resilience of society to food and water crises [21, 22].

What is the impact of migrants on the VWT, and how this interconnects with the water scarcity issue, is still an unexplored topic, although strongly motivated by the water-food-population nexus. Migrants strengthen commercial links among origin and destination countries [4, 23], because of the persistence of alimentary habits outside the country of origin and the expected wealth improvement in the destination country. This affects the trade of food (and water) among countries.

This paper analyzes the relation between the VWT network and the human migration network, with the aim of addressing the following research question: *are migrants beneficial or detrimental to the water endowments of water scarce countries*? To address such question and disentangle the relation between water resources, trade and migration, we first quantify the effect of migration on trade by means of a gravity model exercise, which is functional to model the virtual water fluxes ascribable to each migrant. Then, we evaluate the global and local variations of water footprint (i.e., the water “embedded” in locally-consumed goods) due to migration and finally compare such variations with the migration-induced virtual water trade. The argument is that migration causes an offset of water usage, shifting from an agricultural use of water for locally-consumed food to a use for export towards the countries where migrants are relocated. Therefore migration may result detrimental for the local water endowments. The paper is structured as follows: the first Section describes the data used and defines the methodological framework employed in the study; the following Section illustrates and discusses the results of the empirical investigation, while the last Section discusses the policy implications and draws some concluding remarks.

## Materials and Methods

### Data

The analysis focuses on human migration data (*M*) taken from the United Nation database [24]. Such data express the stock of migrants born in one country, *i*, and living in another country, *j*, considering a total of 232 world countries; data are available for every decade from 1960 to 2010, and for year 2013; we use here data for years 1990, 2000, and 2010, as they overlap with available trade data.

Virtual water trade data (VW) are reconstructed using commodity trade data from the Food and Agricultural Organization of the United Nations [25] and include the international trade of 309 agricultural commodities exchanged from country of origin, *i*, to country of destination, *j*. Annual trade data are available from 1986 to 2010 for 253 countries (cumulating all active countries within such period) [16, 26]. Trade data for each commodity are converted into virtual water flows multiplying them by the water footprint of the commodity in the country of origin of the trade. Water footprints include green and blue water and express the volume of soil water from rainfall and surface-groundwater sources necessary for the growth and production of a unitary weight of each commodity. Data are provided by the global assessment of the Water Footprint Network [27, 28]. The virtual water flows associated to the commodities are then summed to give the total virtual water flows associated to trade; such dataset has been employed in previous works (e.g., [16, 26]). Annual data are averaged over the years 1986-1990, 1991-2000 and 2001-2010 to match the timing of migration data.

Other variables involved in the analysis include the *population*, *x*_*p*_, and the *per capita GDP* (Gross Domestic Product) in USD, *x*_*gdp*_, of all countries over the considered period (1986 to 2010). Data are extracted from the United Nations Statistics Division [29]. Annual data are averaged over the years 1986-1990, 1991-2000 and 2001-2010. In addition, a series of bilateral geographic and economic variables are used as extracted from the CEPII database [30, 31]. Variables include [32] the population-weighted distance between countries, *x*_*d*_, and a series of binary dummy variables, namely *contiguity*, *x*_*c*_ (equal to 1 if the pair shares a common border), *common currency*, *x*_*cc*_ (equal to 1 if the pair shares the currency), *common language*, *x*_*cl*_ (equal to 1 if the pair has the same official language), *colony*, *x*_*col*_ (equal to 1 if one of the two countries is a colony of the other), *regional trade agreements*, *x*_*rta*_ (equal to 1 if the two countries share some regional free trade agreement), *tariff and trade agreements*, *x*_*tta*_, equal to 1 if the given country belong to General Agreement on Tariffs and Trade (GATT) of the World Trade Organization (WTO). The weighted distance and the dummy geographic variables do not depend on time, while dummy economic variables (*rta* and *tta*) are taken for the years 1990, 2000 and 2006, the latter taken as a proxy for year 2010.

### The gravity model

We use a gravity equation to model the relation between human migration and virtual water fluxes. Since the seminal contribution by Tinbergen [33] and Linneman [34], gravity models are extensively used in International Economics to explain trade fluxes [35, 36]. The generalized expression of a gravity model recalls the law of universal gravitation which, after log-transforming the variables, can be managed as a multivariate linear regression.

In our application we include, beside migration data, also population, per capita GDP and dummy variables expressing potential barriers or incentives to trade flows [37–39], so that the baseline gravity model is written as follows:

$$\begin{array}{ccc} {\hat{VW}}_{ij} & = & 10^{b_{0}}\cdot M_{ij}^{b_{1}}\cdot x_{gdp,i}^{b_{2}}\cdot x_{gdp,j}^{b_{3}}\cdot x_{p,i}^{b_{4}}\cdot x_{p,j}^{b_{5}}\cdot x_{d,ij}^{b_{6}}\cdot 10^{\wedge }(b_{7}\cdot x_{c,ij}+\ldots \\ & & +b_{8}\cdot x_{cl,ij}+b_{9}\cdot x_{col,ij}+b_{10}\cdot x_{cc,ij}+b_{11}\cdot x_{rta,ij}+b_{12}\cdot x_{tta,i}+b_{13}\cdot x_{tta,j})\;. \end{array}$$ (1)

${\hat{VW}}_{ij}$ identifies the modeled virtual water flow from country *i* to country *j*, and *b*_0_, *b*_1_, …, *b*_13_ are the regression coefficients estimated with the ordinary least square (OLS) method. Logarithms (log10) are applied to all quantitative variables (dummy variables are not) in order to obtain a desirable additive form of the gravity model, as in the literature for trade [35, 38, 40].

Beside the baseline model, we also apply a set of alternative models, such as the gravity model with Fixed Effects (FE), which is an effective tool from the economic literature [41], to provide robustness to the findings of the proposed analysis. Alternative models as well as some specific hypotheses regarding the water-trade-migration nexus are tested and detailed in the Supporting Information material (S1 Text) and recalled at the end of the following section.

### The water suitcase of migrants

In order to characterize the water-food-migration nexus we propose to quantify the virtual water flow ascribable to each migrant, that we call “*suitcase*”. This measure represents the volume of water that any additional migrant is virtually carrying from his/her origin country to his/her destination country, due to trade flows with the motherland induced by migrant communities.

The variation of a virtual water flux, $\delta \hat{VW}$, associated to a variation of migration, *δM*, (between countries *i* and *j*) can be evaluated with a Taylor expansion to the first order around actual conditions and reads

$$\begin{array}{c} \delta \hat{VW}=b_{1}\cdot \frac{\hat{VW}}{M}\cdot \delta M\;\;, \end{array}$$ (2)

where *b*_1_ is the coefficient of migration in the baseline model, estimated with the OLS method after retaining only significant variables at a 0.1% level with the *t*-Student test. Averaging the above expression over all links having both a virtual water exchange and a non-null migration, the variation of virtual water flux per unit variation of migration, *S*, can be determined as

$$\begin{array}{c} S=\frac{1}{M_{tot}}\sum_{w=1}^{n}M_{w}\frac{\delta \hat{VW}}{\delta M}{|}_{w}=b_{1}\cdot \frac{{\hat{VW}}_{tot}}{M_{tot}}\;\;, \end{array}$$ (3)

where *n* is the overall number of links between countries, ${\hat{VW}}_{tot}$ is the sum of all modeled virtual water fluxes and *M*_*tot*_ is the total number of migrants worldwide. The variable *S* represents the volume of virtual water exchanged worldwide as driven by a unitary increase of migration, thus associated to each migrant as a sort of virtual water suitcase.

Eq (3) can also be referred to one single country at a time, separating incoming fluxes and outgoing fluxes. We define a country-specific virtual water import per immigrant, *S*_*in*_(*x*), and a country-specific virtual water export per emigrant, *S*_*out*_(*x*), which read

$$\begin{array}{c} S_{in}\left(x\right)=b_{1}\cdot \frac{\sum_{w=1}^{n_{in}\left(x\right)}{\hat{VW}}_{in}\left(x\right)}{\sum_{w=1}^{n_{in}\left(x\right)}M_{in}\left(x\right)}\;,\;\;\;\;\;S_{out}\left(x\right)=b_{1}\cdot \frac{\sum_{w=1}^{n_{out}\left(x\right)}{\hat{VW}}_{out}\left(x\right)}{\sum_{w=1}^{n_{out}\left(x\right)}M_{out}\left(x\right)}\;, \end{array}$$ (4)

respectively, where the subscript _*in*_ identifies links and fluxes directed towards country *x* and the subscript _*out*_ identifies links and fluxes directed away from country *x*. *S*_*in*_(*x*) quantifies the country-specific virtual water import associated to each foreigner living within the country *x* and *S*_*out*_(*x*) quantifies the country-specific virtual water export associated to each person who left country *x* and lives abroad.

## Results and discussions

### The relationship between virtual water trade and migrations

Human migration induces an intensification of trade flows, which in turn implies an increased virtual water flow between the motherland and the destination country. A first clue of the relevance of migrations for determining the VW flows is provided by the correlation coefficient between the two variables, which is about 0.5 in 2010, and is visually confirmed by the scattered representation of VW flows and migration fluxes (S1 Fig).

The relation between virtual water flows and migration could also derive from the dependence of the two variables on other descriptors, as correlation does not imply causation. To investigate this issue, we collected data for several explanatory variables, and applied a multivariate regression model (or gravity model, as in Eq (1)). The wideness of the controls used in the regression, which covers economic, geographical, demographic and political aspects, aims to properly isolate the causal effect of migrants on VW trade while reducing all sources of omitted variable bias.

Migrations turn out to be determinant in explaining the VW flows also in the presence of all other variables, with OLS-estimated regression coefficients detailed in S1 Table and results are found to be robust in time. These findings are in line with other studies on the relationship between migrations and trade in terms of economic values [5]. The key role of migration is also confirmed by our robustness checks. The FE gravity model specification produces similar results in terms of migration coefficients (Section A in S1 Text). Alternative models based on count process, traditionally used to better cope with a large amount of zero flows, such as Poisson, negative binomial, and zero inflated Poisson, also show positive and significant coefficients for migration, which are in line with the OLS and FE results (Section A in S1 Text).

Migration coefficients of gravity models are growing in time, possibly indicating an increasing relevance of migrations. However, given the temporal evolution of the VW trade network [26], a more rigorous indication of the role of migration in explaining VW flows can be given by a commonality analysis [42]. Commonality analysis partitions the variance of modeled VW flows into the contribution given by each variable in the multivariate linear regression. The analysis (Section B in S1 Text) confirms the relevance of migrations in explaining VW flows across the trade network, and the increasing role of migrations in time. Further analyses support the following evidence:

- Migration-induced VW trade is more evident in the trade of crops and animal-based commodities, while migrants have less importance in explaining VW trade associated to luxury food and non edible commodities (Section C in S1 Text).
- The effect of migration fluxes, i.e. migrants moving in a certain period, on the VW trade is significant, but lower in magnitude compared to stocks of migrants. This is confirmed by the comparison of regression results between migration fluxes and migration stocks (Section D in S1 Text) that could be justified by a temporal delay between the migrants settlement and trade initiation/intensification.
- Causation, besides correlation, characterizes the relation between human migration and VW trade. An instrumental variable approach allowed us to remove reverse causality of migrations on VW trade flows (Section E in S1 Text).
- VW trade is not significantly affected by the movement of refugees and asylum seekers, a peculiar case of migration (Section F in S1 Text). They leave their motherland often for socio-economical or political shocks and they are unlikely to foster the export of food from origin countries.

### The water suitcase of migrants

The gravity model allows us to quantify the volume of virtual water exchanged worldwide as driven by a unit increase of human migration, i.e. the water suitcase of migrants, as defined in Materials and Methods section. Using the migration coefficient, *b*_1_, shown in Table 1, we find that the global average suitcase increases from 233 m^3^ per capita per year in 1990 to 1367 m^3^ per capita per year in 2010 with a tendency to double for every decade. The evolution towards larger water suitcase and the increasing number of migrants worldwide suggests an overall expansion of the total volume of virtual water associated to (and driven by) migration. Such increase is, likely, justified by both the overall increase of virtual water volumes exchanged worldwide [26] and by the increasing role of migration in determining trade.

**Table 1:** Results overview. Gravity model migration coefficient, *b*_1_, estimated using only significant regressors at a 0.1% level with a t-student test. “WF” refers to the per-capita water footprint of consumption.

Measure	1990	2000	2010
Migration coefficient, *b*_1_	0.098	0.185	0.306
Average suitcase of migrants [m^3^/p/y]	233.47	696.73	1366.7
WF of migrants in motherland [km^3^/y]	144.5	231.3	304.3
WF of migrants in destination countries [km^3^/y]	180.6	288.0	399.5

**Fig 1:**
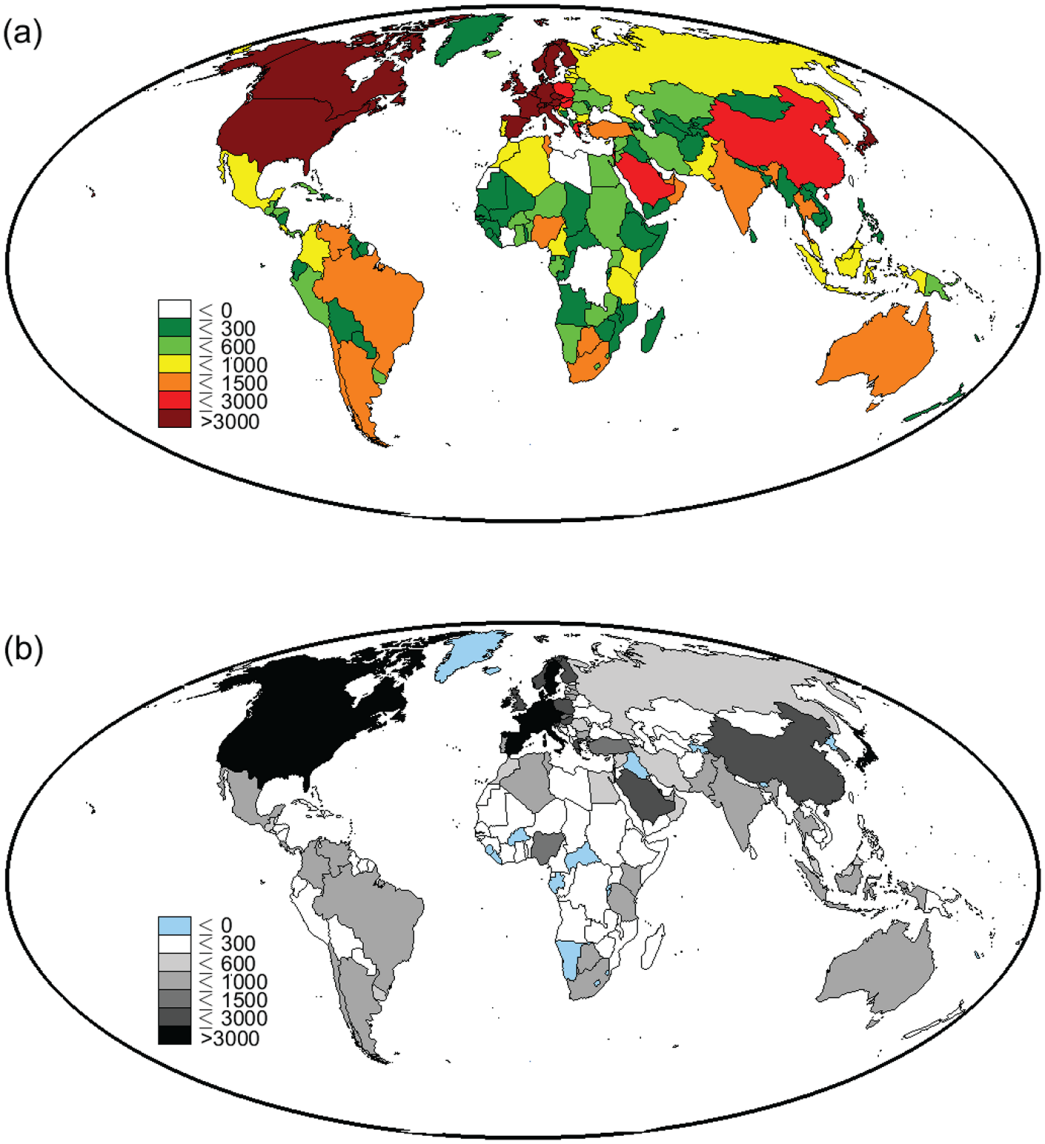
World map of the water suitcase. (a) The water suitcase of emigrants from each country in 2010 and (b) water suitcase variations from 1990 to 2010; suitcases and variations are measured in m^3^ per capita per year.

In order to characterize the geography of virtual water suitcases by country, one may focus on outgoing fluxes and define the water suitcase of emigrants, *S*_*out*_, quantifying the country-specific virtual water export associated to each person who left the country and lives abroad. The map of emigrant suitcases in 2010 and their temporal variations from 1990 to 2010 are shown in Fig 1. Likewise, a country-specific suitcase of immigrants can be defined using virtual water imports (S2 Fig), but it appears less relevant for the considerations that follow.

People leaving North American and Central European countries have a larger suitcase, likely depending on the advanced socio-economic conditions and living standards of emigrants from these countries and their capability of inducing trade when moving abroad. The virtual water suitcase of emigrants, in fact, appears to be correlated to the socio-economical wealth of the country of origin, with the correlation coefficient between the suitcase and the per-capita GDP being 0.325 in 1990 and 0.527 in 2010. People from major Asian countries (except Japan and China) have a small suitcase when leaving their home countries, similarly to some Central African and Near East regions.

Temporal trends are investigated by taking the difference between the suitcase of emigrants in the 2010 and in the 1990 decade. The map of differences confirms and details the marked increase anticipated above (Table 1) for global values. The overall trend from 1990 to 2010 is clear: countries already having large outgoing suitcases in 1990, such as North American and European countries, have further increased the suitcase values. Emerging economies are those showing a very high increase from 1990 to 2010; examples include China, Turkey, Brazil, Argentina and Australia, while African countries maintain their heterogeneity, still showing a clear increasing pattern. Cases without VW increases involve countries with limited export (e.g., New Zealand, Mongolia, Kazakhstan) or undergoing economical-political crises in the period examined (e.g., Afghanistan, Iraq, Somalia and Central African Republic).

### The water footprint of migrations

We now consider the specific impact of migration on the water resources of home countries. To address this point, we employ the per-capita water footprint (WF) of consumption, that is the virtual water content of all goods consumed within a country divided by the population in the country. Averaging over the period 1996-2005, Hoekstra and Mekonnen [7] found a global average of 1385 m^3^/y per capita, and highly heterogeneous values across countries [43].

The global water footprints of migrants in origin and destination countries are first compared. To this purpose, migration stocks are multiplied by the per-capita WF of people in the two countries [43] and summed up over all migration links worldwide. The global WF of migrants in destination countries in 2010 was 400 km^3^/y, which compares with a global WF in motherland of 304 km^3^/y. The difference between the two volumes represents the gross impact of migration on global water resources and it has doubled in 20 years, as shown in Table 1. The increasing gross impact is due to the marked increase of migrating people and to the evolution of the migration network; in fact, the impact grew in time at a more-than-proportional rate than migration even if the country-specific per-capita WF is taken constant in this analysis.

The global WF in destination countries is larger than in the home countries, likely due to the fact that major migration fluxes occur from poorer to richer countries [44], thus (likely, although not systematically) from countries having a lower per-capita water footprint to countries with higher per-capita water footprint. Therefore migration appears to enhance the pressure on global water resources. Although being approximations not accounting for relevant factors, such as the socio-economical conditions of immigrants in destination countries and their rapidity of adaptation to local diets, these volumes give an indicative measure of the increase of virtual water consumption associated to migration.

**Fig 2:**
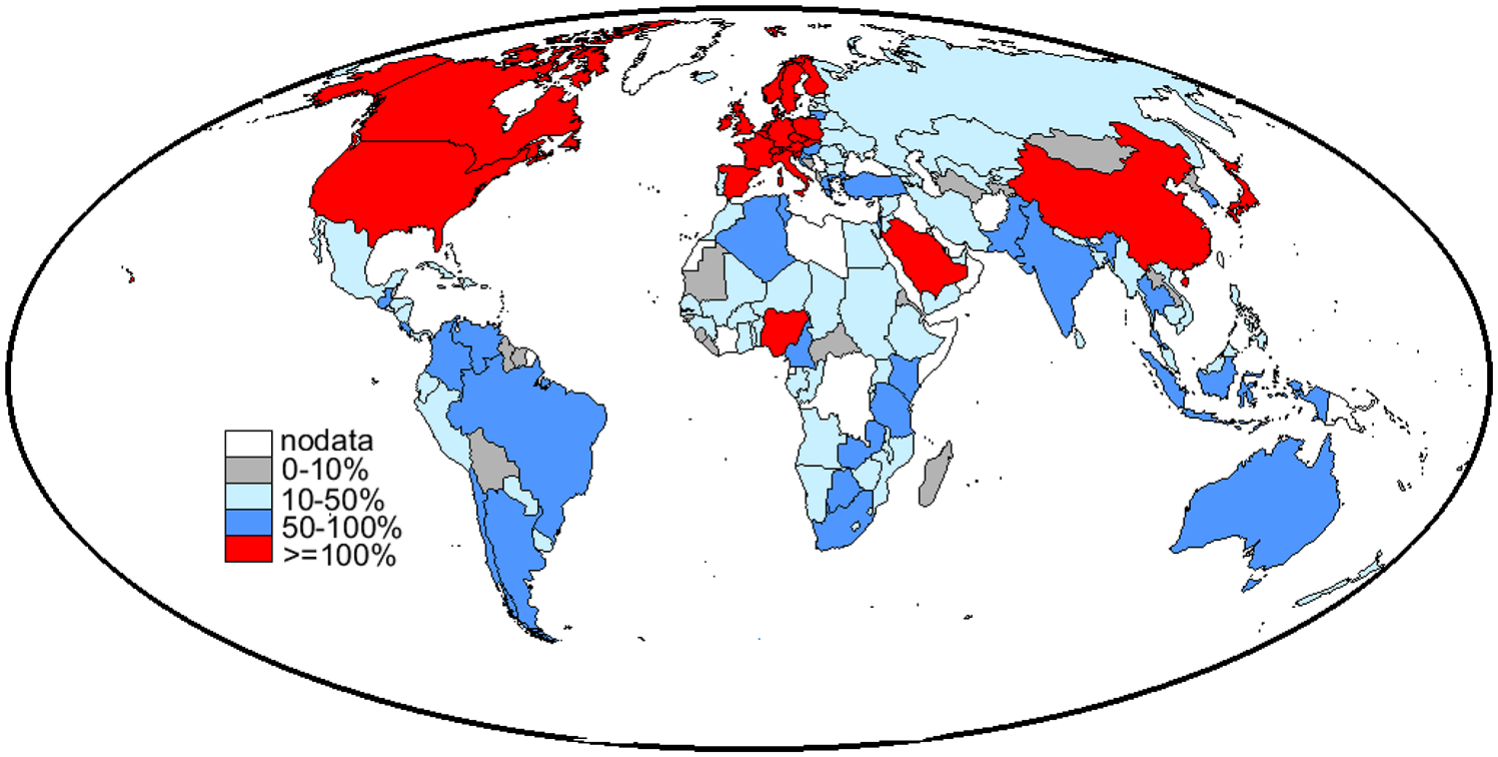
World map of the ratio between water suitcase of an emigrant and WF of an inhabitant, in decade 2010. The percentage ratio quantifies the water pressure relief (in red) or increase (in blue) in each country due to migrants having left the country.

However, the net impact of migration on global water resources should account for the increased demand of goods in motherlands due to trade intensification between the origin and destination countries, i.e. the water suitcase of migrants. The water suitcase of people leaving each country is, thus, compared to the per-capita WF of motherland [43]. The percentage ratio between the two variables is represented in Fig 2 and shows which countries benefit from migrant expatriation (ratio ≤ 100%), and which countries don’t (ratio ≥ 100%). In most countries the water suitcase of emigrants is lower or comparable to the water footprint of inhabitants, thus migration induces a water pressure relief, while in few countries (North America, most of Europe, Saudi Arabia, Nigeria, China and Japan) the water suitcase of emigrants is larger than the water footprint of inhabitants. In the latter case, emigrants are further depleting the water resources of their motherland to meet the virtual water demand for food production associated to migration-induced trade. A comparison with the same percentage ratio in 2000 (S3 Fig) shows an average increase of the ratio in the last decade, corresponding to a detrimental effect of migration on water endowments. In particular, in China, Saudi Arabia and Nigeria, as well as in the south of Europe (Italy and Spain), the suitcases of emigrants were smaller than the per-capita WF in the country of origin in 2000, while turned out to be higher in 2010. Most Asian and African water-stressed countries also increased the value of the ratio, giving a confirmation of the increasing role played by migrants in negatively affecting the countries’ water pressures.

### The network of migration impacts

We investigate more in depth the countries where the (per-capita) water suitcase of emigrants is higher than the water footprint of inhabitants, using network analysis techniques. For each pair of countries where a flux of VW and migrants is active, we calculate the migration impact associated to such link as the difference between the per-capita water footprint in the origin country and the water suitcase of each emigrant, multiplied by the number of migrants on that link.

**Fig 3:**
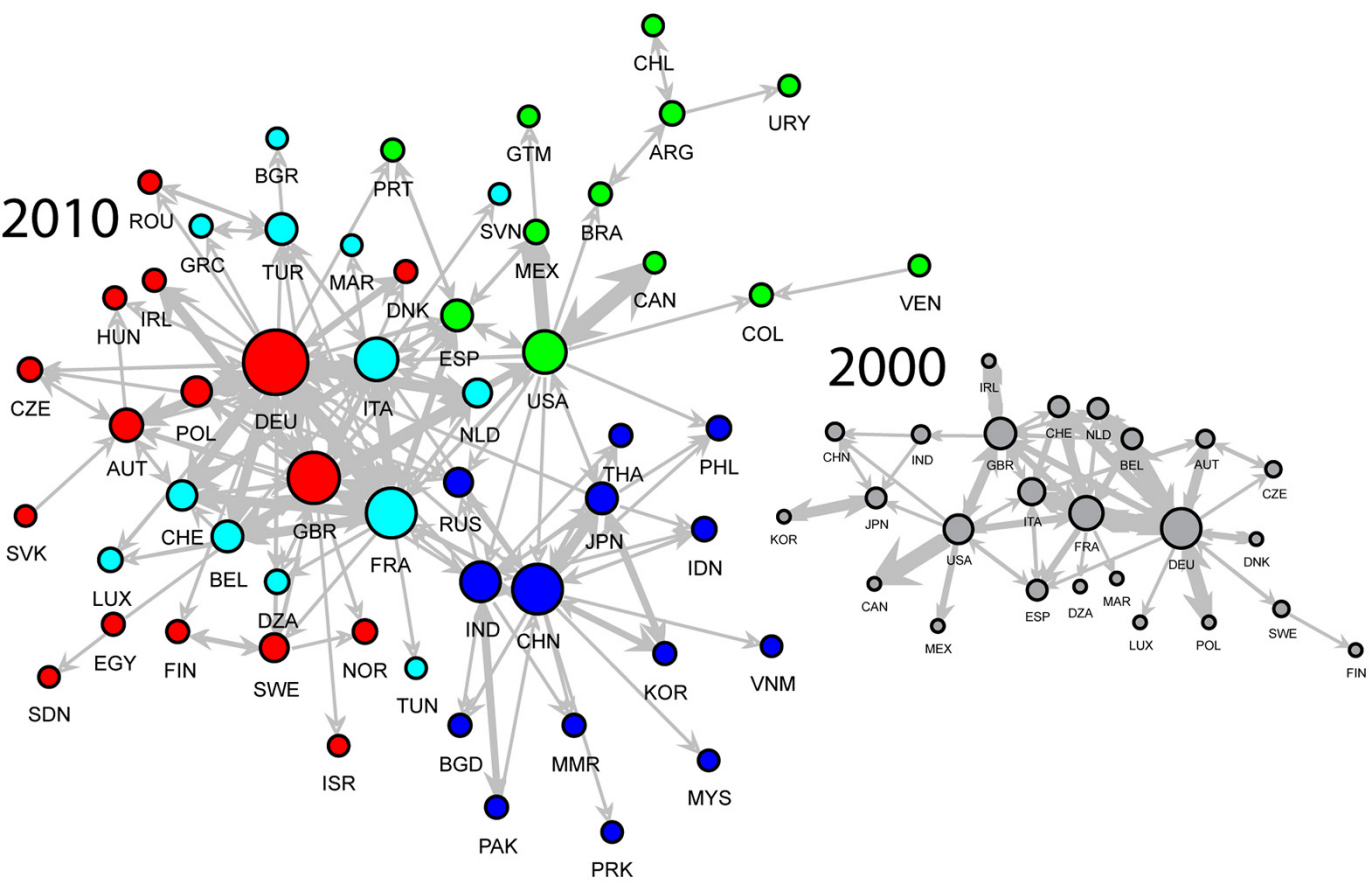
Network structure of migration impacts on water resources. The network represents the countries (nodes) and the corresponding migrations (links) where we find a negative migration impact in 2010 (2000 in the inset).

We consider only the links where the migration impact is negative, i.e. migrations are detrimental to the water resources of origin countries. Results in the left chart of Fig 3 use nodes’ dimensions to identify country’s centrality and the different colors of nodes to represent communities according to the Newman-Girvan community detection algorithm. Evidences found in Fig 2 are confirmed: USA, China and Central European countries are the most central regions in the network. Furthermore, Fig 3 highlights that emigrants from those countries have a bigger suitcase (relative to footprint) when moving towards several destinations. Comparison with the network in 2000 also highlights an evident increase in the number of country pairs with a negative migration impact. Emerging countries (the blue cluster in the network), such as India and China, are now more central in the network.

## Conclusions and policy recommendations

Virtual water trade provides water-scarce countries a chance to minimize internal water use by relying on imports of food products from water-rich countries, thus alleviating water scarcity problems through global economic processes. Yet, currently the global water savings envisaged in virtual water trade analyses is happening to a limited extent: Hoekstra [45] finds that international trade is reducing global water-use in agriculture by 5%; Costinot et al. [46] find that trade in agricultural products mainly conforms to the rules of comparative cost advantages rather than to a global optimization of resource use.

In recent empirical analyses, it is said that the movement of production factors (such as migrants) across borders induces more trade [5], and this phenomenon is relevant also for VW trade, triggering the discussion about its causes, and possible policy implications. We have added here the human-migration dimension to such analyses, investigating whether migrants from water-scarce countries are beneficial or detrimental to water endowments of their motherland. We have thus measured the volume of the additional virtual water flow out of the countries of origin as associated to each migrant (the water suitcase of migrants), and compared it to the water footprint of origin countries. Overall, we find that the impact on the water pressure associated to migration is heterogeneous over countries. However, in several countries, this impact turns negative over the time: i.e., the expatriation-induced trade is so much relevant to induce a detrimental effect of migrations on the water resources of the motherland.

As pointed out by Kelley et al. [47] for the (different) relation between climate, food production and human migrations, the role of institutions and government policies (particularly, sustainable agricultural and environmental policies) is pivotal for preventing global catastrophes. Similarly, a key role has to be played by government policies for preventing that human migrations further stress water resources of their arid and semi-arid countries of origin. Countries should therefore adopt appropriate policies aimed at reducing such virtual water flows, especially when detrimental to the water endowments of severely water-scarce countries. For instance, origin countries could adopt specific monitoring mechanisms for tracking the exports of those water-intensive products which are more likely to be part of the diet preferences of migrants, eventually putting in place WTO-compatible trade policy measures aimed at preventing the depletion of their water resources for the most worrisome trade flows. Examples of such measures may range from products’ water-content labeling schemes to regulatory measures such as (environmental) processes and production methods. Alternatively, origin countries could use the water pricing leverage when allocating water use’ rights to those worrisome productions (taking into account and calibrating with the related income effects upon the affected population).

Furthermore, host and origin countries could co-operate in implementing policies to enhance the role that return migrants can play to reduce the water footprint of food production in their countries of origin, for example allowing for increased transfer of technologies and production methods to the countries of origin. Host countries could play a role for enhancing migrants exposure to (and learning of) water-efficient production technologies and methods, as well as access to technologies for reducing water losses from leakages in the infrastructure and reservoirs or for improving waste water treatments. Countries of origin could facilitate the application of these technologies from the return migrants, providing for example targeted incentives. Such actions may contribute to reduce the water footprint of domestic production in the countries of origin, as well as to reduce the water suitcase of the successive migrants.

## Supporting Information

S1 Fig Scatter plot. Scatter plot between virtual water flows and migration fluxes, in decade 2010.
(EPS)

S2 Fig Water suitcase of immigrants in 2010. World map of the water suitcase of immigrants in decade 2010, measured in m^3^ per capita per year.
(TIF)

S3 Fig Water relief in decade 2000. World map of the percentage ratio of the water suitcase of emigrants in decade 2000 over the water footprint of inhabitants, measured in m^3^ per capita per year.
(TIF)

S1 Table Baseline gravity models results. Gravity model results obtained with the ordinary least square (OLS) method considering only links, or directed country pairs, which are active on both VW trade and migration. Variables are explained in the **Data** section of the paper, “rta” stands for *regional trade agreements* and “tta” stands for *tariff and trade agreements*. Notations following the coefficient values identify the level of significance, *p*, of the considered variable at a *t*-Student test, i.e. ‘*’ (*p* < 0.05), ‘**’ (*p* < 0.01), ‘***’ (*p* < 0.001); “N. observations” identifies the number of links considered in the model calibration, and “*R*^2^-adjusted” is the coefficient of determination of the model output, adjusted by the number of calibrated parameters.
(PDF)

S1 Text Additional elaborations and results. Methodological and quantitative support to some results mentioned in the paper.
(PDF)

S1 File Dataset for the gravity models. Tab-delimited *txt* file containing all variables employed in the gravity models for decades 1990, 2000 and 2010.
(TXT)

## References

[1] FAO. Report of the 32nd Regional Conference for the Near East; 2014.

[2] Davis KF, D’Odorico P, Laio F, Ridolfi L. Global spatio-temporal patterns in human migration: a complex network perspective. PLoS ONE. 2013;8(1). doi: 10.1371/journal.pone.0053723 PMC3553122 23372664

[3] Fagiolo G, Mastrorillo M. International migration network: Topology and modeling. Phys Rev E. 2013;88. doi: 10.1103/PhysRevE.88.012812 23944523

[4] Sgrignoli P, Metulini R, Schiavo S, Riccaboni M. The relation between global migration and trade networks. Physica A: Statistical Mechanics and its Applications. 2015;417:245–260. doi: 10.1016/j.physa.2014.09.037

[5] Genc M, Gheasi M, Nijkamp P, Poot J. The impact of immigration on international trade: a meta-analysis1. Migration Impact Assessment: New Horizons. 2012;p. 301. doi: 10.4337/9780857934581.00019

[6] Falkenmark MJ, Rockström J. Balancing water for humans and nature: The New Approach in Ecohydrology. Earthscan, London; 2004.

[7] Hoekstra AY, Mekonnen MM. The water footprint of humanity. PNAS. 2012;109(9):3232–3237. doi: 10.1073/pnas.1109936109 22331890 PMC3295316

[8] Allan JA. Virtual water: A strategic resource global solutions to regional deficits. Groundwater. 1998;36(4):545–546. doi: 10.1111/j.1745-6584.1998.tb02825.x

[9] Reimer JJ. On the economics of virtual water trade. Ecological Economics. 2012;75:135–139. doi: 10.1016/j.ecolecon.2012.01.011

[10] Davis DR, Weinstein DE. Market access, economic geography and comparative advantage: an empirical test. Journal of International Economics. 2003;59(1):1–23. doi: 10.1016/S0022-1996(02)00088-0

[11] Hoekstra AY, Hung PQ. Virtual water trade: A quantification of virtual water flows between nations in relation to international crop trade. UNESCO-IHE, Delft, the Netherlands; 2002.

[12] Lenzen M. Understanding virtual water flows: A multiregion input-output case study of Victoria. Water Resour Res. 2009;45. doi: 10.1029/2008WR007649

[13] Konar M, Dalin C, Suweis S, Hanasaki N, Rinaldo A, Rodriguez-Iturbe I. Water for food: The global virtual water trade network. Water Resour Res. 2011;47. 10.1073/pnas.1203176109 PMC3341016 22474363

[14] D’Odorico P, Carr JA, Laio F, Ridolfi L. Spatial organization and drivers of the virtual water trade: A community-structure analysis. Environmental Research Letters. 2012;7(3):034007. doi: 10.1088/1748-9326/7/3/034007

[15] Carr J, D’Odorico P, Laio F, Ridolfi L. Recent history and geography of virtual water trade. PLoS ONE. 2013;8(2). doi: 10.1371/journal.pone.0055825 PMC3574060 23457481

[16] Tamea S, Allamano P, Carr JA, Claps P, Laio F, Ridolfi L. Local and global perspectives on the virtual water trade. Hydrol Earth Syst Sci. 2013;17. doi: 10.5194/hess-17-1205-2013

[17] Sartori M, Schiavo S. Connected we stand: A network perspective on trade and global food security. Food Policy. 2015;57:114–127. doi: 10.1016/j.foodpol.2015.10.004

[18] Godfray HCJ, Beddington JR, Crute IR, Haddad L, Lawrence D, Muir JF, et al. Food security: The challenge of feeding 9 billion people. Science. 2010;327:812–818. doi: 10.1126/science.1185383 20110467

[19] Rosegrant M, Cai X, Cline S. World water and food to 2025: Dealing with scarcity. International Food Policy Research Institute [IFRI], Washington DC; 2002.

[20] Chapagain AK, Hoekstra AY, Savenije HHG. Water saving through international trade of agricultural products. Hydrol Earth Syst Sci. 2006;10:455–468. doi: 10.5194/hess-10-455-2006

[21] Seekell DA, D’Odorico P, Pace ML. Virtual water transfers unlikely to redress inequality in global water use. Environ Res Lett. 2011;6. doi: 10.1088/1748-9326/6/2/024017

[22] Porkka M, Kummu M, Siebert S, Varis O. From food insufficiency towards trade dependency: A historical analysis of global food availability. PLoS ONE. 2013;8(12). doi: 10.1371/journal.pone.0082714 24367545 PMC3867377

[23] Rauch JE. Business and social networks in international trade. Journal of economic literature. 2001;p. 1177–1203. doi: 10.1257/jel.39.4.1177

[24] United Nations. Trends in International Migrant Stock: The 2013 Revision - Migrants by Destination and Origin, database POP/DB/MIG/Stock/Rev.2013/Origin; 2013. Accessed on 12/11/2013. Available at http://www.un.org/en/development/desa/population/migration/data/

[25] FAO. FAOSTAT database; 2012. Accessed on 07/10/2012. Available at http://faostat3.fao.org/home/index.html

[26] Carr J, D’Odorico P, Laio F, Ridolfi L. On the temporal variability of the virtual water network. Geophys Res Lett. 2012;39. doi: 10.1029/2012GL051247

[27] Mekonnen MM, Hoekstra AY. The green, blue and grey water footprint of crops and derived crop products. UNESCO-IHE, Delft, the Netherlands; 2010. 47. Appendix II.

[28] Mekonnen MM, Hoekstra AY. The green, blue and grey water footprint of farm animals and animal products. UNESCO-IHE, Delft, the Netherlands; 2010. 48. Appendix V.

[29] Nations U. National Accounts Main Aggregate Database; 2013. Accessed on 18/11/2013. Available at http://unstats.un.org/unsd/snaama/dnllist.asp

[30] CEPII. GeoDist database; 2013. Accessed on 12/11/2013. Available at http://www.cepii.fr/cepii/en/bdd_modele/presentation.asp?id=6

[31] CEPII. Gravity database; 2010. Accessed on 23/9/2010. Available at http://www.cepii.fr/CEPII/en/bdd_modele/presentation.asp?id=8

[32] Mayer T, Zignago S. Notes on CEPII distances measures: The GeoDist database. CEPII working paper; 2011.

[33] Tinbergen J. Shaping the world economy; suggestions for an international economic policy. Twentieth Century Fund, New York; 1962.

[34] Linnemann H. An econometric study of international trade flows. North-Holland Publishing Company, Amsterdam; 1966.

[35] Frankel J, Rose A. An estimate of the effect of currency unions on trade and growth. NBER Working Paper. 2000;7857.

[36] Egger P. An econometric view on the estimation of gravity models and the calculation of trade potentials. The World Economy. 2002;25(2):297–312. doi: 10.1111/1467-9701.00432

[37] Baier SL, Bergstrand JH. Do free trade agreements actually increase members’ international trade? Journal of international Economics. 2007;71(1):72–95. doi: 10.1016/j.jinteco.2006.02.005

[38] Glick R, Rose AK. Does a currency union affect trade? The time-series evidence. European Economic Review. 2002;46(6):1125–1151. doi: 10.1016/S0014-2921(01)00202-1

[39] Bun MJ, Klaassen FJ. The Euro Effect on trade is not as large as commonly thought. Oxford Bulletin of Economics and Statistics. 2007;69(4):473–496. doi: 10.1111/j.1468-0084.2007.00448.x

[40] Feenstra RC, Markusen JR, Rose AK. Using the gravity equation to differentiate among alternative theories of trade. Canadian Journal of Economics/Revue canadienne d’économique. 2001;34(2):430–447. doi: 10.1111/0008-4085.00082

[41] Harrigan J. Openness to Trade in Manufactures in the OECD. Journal of international economics. 1996;40(1):23–39. doi: 10.1016/0022-1996(95)01395-4

[42] Newton R, Spurrell D. Examples of the use of elements for clarifying regression analyses. Applied Statistics. 1967;p. 165–172. doi: 10.2307/2985778

[43] Mekonnen MM, Hoekstra AY. National water footprint accounts: The green, blue, and grey water footprint of production and consumption. UNESCO-IHE, Delft, the Netherlands; 2011. 50. Appendix VIII.

[44] Nations U. International Migration Report 2013. United Nations (U.N.), Department of Economic and Social Affairs, Population Division; 2013.

[45] Hoekstra AJ. The relation between international trade and freshwater scarcity. WTO Staff Working Paper; 2010.

[46] Costinot A, Donaldson D, Komunjer I. What goods do countries trade? A quantitative exploration of Ricardo’s ideas. The Review of Economic Studies. 2012;79(2):581–608. doi: 10.1093/restud/rdr033

[47] Kelley CP, Mohtadi S, Cane MA, Seager R, Kushnir Y. Climate change in the Fertile Crescent and implications of the recent Syrian drought. Proceedings of the National Academy of Sciences. 2015;112(11):3241–3246. doi: 10.1073/pnas.1421533112 PMC4371967 25733898

