# Supplementary figures and images for: The Water Suitcase of Migrants: Assessing Virtual Water Fluxes Associated to Human Migration
Source: PLoS One. 2016 Apr 28;11(4):e0153982. doi: 10.1371/journal.pone.0153982 (PMC4849658; doi:10.1371/journal.pone.0153982)

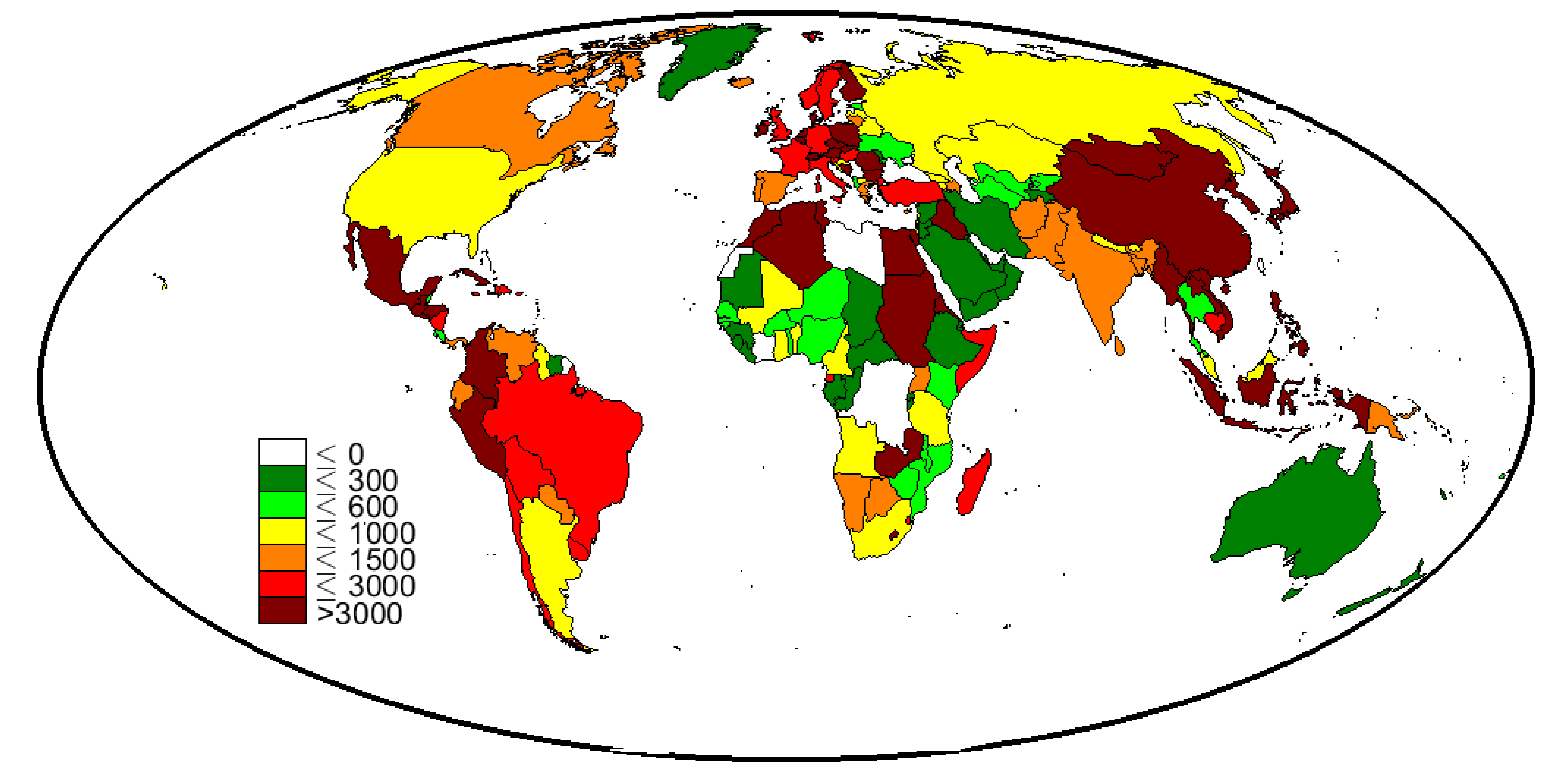

Supplement: S2 Fig — World map of the water suitcase of immigrants in decade 2010, measured in m3 per capita per year. (TIF) [file pone.0153982.s002.tif]

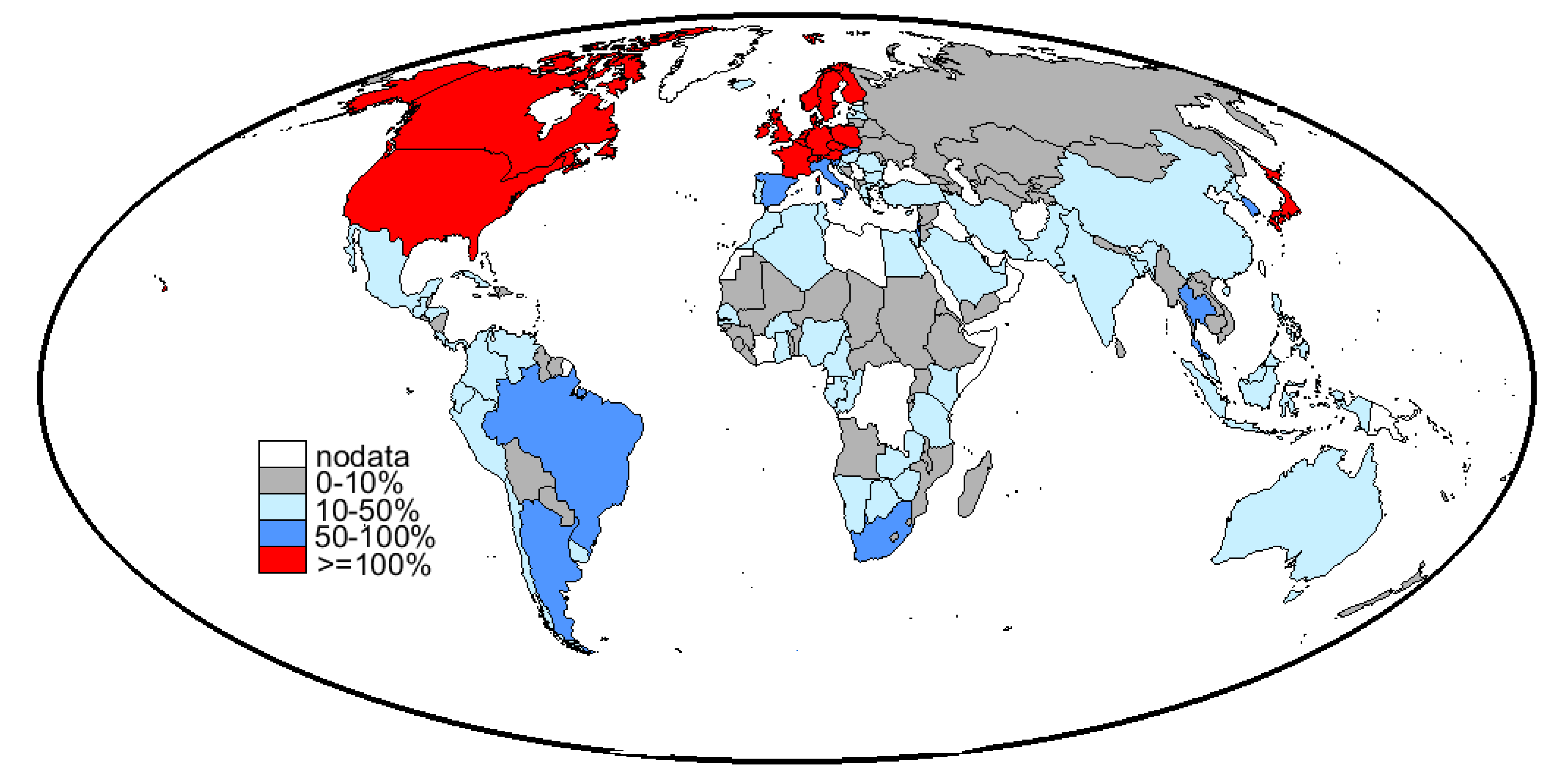

Supplement: S3 Fig — World map of the percentage ratio of the water suitcase of emigrants in decade 2000 over the water footprint of inhabitants, measured in m3 per capita per year. (TIF) [file pone.0153982.s003.tif]
