# Supplementary material for: The Water Suitcase of Migrants: Assessing Virtual Water Fluxes Associated to Human Migration
Source: PLoS One. 2016 Apr 28;11(4):e0153982. doi: 10.1371/journal.pone.0153982 (PMC4849658; doi:10.1371/journal.pone.0153982)
Supplement: S1 Table — Gravity model results obtained with the ordinary least square (OLS) method considering only links, or directed country pairs, which are active on both VW trade and migration. Variables are explained in the Data section of the paper, “rta” stands for regional trade agreements and “tta” stands for tariff and trade agreements. Notations following the coefficient values identify the level of significance, p, of the considered variable at a t-Student test, i.e. ‘*’ (p < 0.05), ‘**’ (p < 0.01), ‘***’ (p < 0.001); “N. observations” identifies the number of links considered in the model calibration, and “R2-adjusted” is the coefficient of determination of the model output, adjusted by the number of calibrated parameters. (PDF) [file pone.0153982.s004.pdf]

**Decade**	**1990**	**2000**	**2010**
**migration**	0.099***	0.185***	0.305***
**weighted distance**	-0.748***	-0.862***	-0.881***
**population (o)**	0.658***	0.836***	0.824***
**population (d)**	0.729***	0.681***	0.658***
**per capita GDP (o)**	0.384***	0.486***	0.402***
**per capita GDP (d)**	0.650***	0.558***	0.370***
**contiguity**	0.267**	0.297***	0.298***
**common language**	0.168***	0.190***	0.263***
**colony**	0.577***	0.469***	0.316 ***
**common currency**	0.440*	-0.140	0.134
**rta**	0.095	0.183***	0.282***
**tta (o)**	0.315***	0.443***	0.258***
**tta (d)**	-0.090	0.003	-0.188***
**N. observations**	5959	8874	9261
R^2**-adjusted**	0.359	0.454	0.483
