# Supplementary material for: The Water Suitcase of Migrants: Assessing Virtual Water Fluxes Associated to Human Migration
Source: PLoS One. 2016 Apr 28;11(4):e0153982. doi: 10.1371/journal.pone.0153982 (PMC4849658; doi:10.1371/journal.pone.0153982)
Supplement: S1 Text — Methodological and quantitative support to some results mentioned in the paper. (PDF) [file pone.0153982.s005.pdf]

Supporting Information (SI) for the paper "The Water Suitcase of Migrants: Assessing Virtual Water Fluxes Associated to Human Migration"

Metulini, R.[1,2,*], Tamea, S.[3], Laio, F.[3], Riccaboni, M.[2,4]

[1]: Scuola Superiore S.Anna - Institute of Economics, Piazza Martiri della Libertà, 33, 56127 Pisa, Italy
[2]: IMT Institute for Advanced Studies Lucca - Laboratory of Innovation Management and Economics, Piazza San Francesco 19, 55100 Lucca, Italy
[3]: Politecnico di Torino - Dept. of Environment, Land and Infrastructure Engineering, Corso Duca degli Abruzzi 24, 10129 Torino, Italy
[4]: KU Leuven - Department of Management, Stategy and Innovation, Naamsestraat 69, 3000 Leuven, Belgium
[*]: r.metulini@sssup.it

S1 Text. Additional elaborations and results.

This supplementary text provides additional information supporting some statements given in the main paper, both form a methodological and quantitative point of view. All variables employed in the following analyses are detailed in the **Data** section of the main paper or within the supplementary text itself. In the tables shown hereafter, "rta" stands for *regional trade agreements* and "tta" stands for *tariff and trade agreements*. Notations following the coefficient values identify the level of significance of the considered variable at a t-Student test, i.e. '*' ($p < 0.05$), '**' ($p < 0.01$), '***' ($p < 0.001$); "N. observations" identifies the number of links, or country pairs, considered in the model calibration, and "R^2-adjusted" is the coefficient of determination of the model output, adjusted by the number of calibrated parameters.

A Alternative model results: the Fixed Effects formulation and other count models

The ordinary least squares (OLS) method has traditionally been used for estimating the coefficients of the baseline gravity specification in its log-linear form. While the base-

line gravity equation (Eq. (1) in the main text) is commonly used in empirical analysis, multilateral trade resistances (MTR) may be taken into account [1]. According to Baier and Bergstrand [2], a simple way of treating MTR is the use of *fixed effect* (FE) model, which was first emphasized by Harrigan [3]. This model specification consists of including origin-specific and destination-specific countries' intercepts in the regression. This approach includes the effect of cross-country variations of explanatory variables but precludes, in cross-sectional regressions, the analysis of country-specific determinants. We thus employ the FE model dropping all country-specific variables but the dummy indicating the belonging of the country to the tariff and trade agreement. The resulting multiplicative model reads as follow:

$$\widehat{VW}_{ij} = 10^{\alpha_i} \cdot 10^{\alpha_j} \cdot M_{ij}^{a_1} \cdot x_{d,ij}^{a_2} \cdot 10^\wedge \big(a_3 \cdot x_{c,ij} + a_4 \cdot x_{cl,ij} + a_5 \cdot x_{col,ij} + \dots \\ + a_6 \cdot x_{cc,ij} + a_7 \cdot x_{rta,ij} + a_8 \cdot x_{tta,i} + a_9 \cdot x_{tta,j}\big) \quad (1)$$

where α_i and α_j are the n-dimension vector of coefficients to be estimated relative to origin and destination countries' fixed effects, respectively, while a_1, a_2,... a_9 are the coefficients related to migration, distance, contiguity, common language, colony, common currency, regional trade agreement, and tariff trade agreement (of origin and destination country), respectively.

Table A.1: Gravity model results obtained with the ordinary least square (OLS) method. Results are estimated considering only active links on both trade and migration.

**Variable / Decade**	**1990**	**2000**	**2010**
**migration**	0.099***	0.185***	0.305***
**weighted distance**	-0.748***	-0.862***	-0.881***
**population (o)**	0.658***	0.836***	0.824***
**population (d)**	0.729***	0.681***	0.658***
**per capita GDP (o)**	0.384***	0.486***	0.402***
**per capita GDP (d)**	0.650***	0.558***	0.370***
**contiguity**	0.267**	0.297***	0.298***
**common language**	0.168***	0.190***	0.263***
**colony**	0.577***	0.469***	0.316***
**common currency**	0.440*	-0.140	0.134
**rta**	0.095	0.183***	0.282***
**tta (o)**	0.315***	0.443***	0.258***
**tta (d)**	-0.090	0.003	-0.188***
**N. observations**	5959	8874	9261
**R^2-adjusted**	0.359	0.454	0.483

Other models are used as a check for results robustness: in particular we apply the Poisson and the Negative Binomial (NegBin) models, the zero-inflated Poisson (ZIP) and

Table A.2: Gravity model results, using the fixed effect (FE) formulation. Results are estimated considering only active links on both trade and migration.

Variable / Decade	1990	2000	2010
**migration**	0.198***	0.282***	0.299***
**weighted distance**	-0.844***	-0.971***	-1.103***
**contiguity**	0.187***	0.229***	0.238***
**common language**	0.150***	0.139***	0.203***
**colony**	0.430***	0.424***	0.361***
**common currency**	0.604***	-0.254***	0.002
**rta**	-0.118	-0.077***	0.149***
**tta(o)**	1.263***	1.755***	1.464***
**tta(d)**	-0.512	-0.225	0.099
**N. observations**	6078	9007	9303
R^2**-adjusted**	0.642	0.673	0.705

zero-inflated Negative Binomial (ZINB). Poisson and NegBin are valid alternatives to OLS, which are based on considering the dependent variable as a count process, removing the assumption of normal distribution and enabling the accounting of a high share of zero flows. In particular, NegBin introduces a parameter, called θ, controlling for distribution's overdispersion. The zero inflated models consist on a two step procedure. In the first step, we estimate the probability of existence of a link between two countries (at least one m^3 of VW) with a *logit* regression. In the second step we estimate the value of VW with a Poisson or NegBin model, conditional to the first step. The ZIP and ZINB permits to obtain consistent estimates even when big amount of zeros are present.

B Commonality analysis

Considering a multivariate regression between a dependent variable Y and a set of regressors x_1,x_2,x_3, ..., the variance of Y can be expressed partly by the contribution of each regressor alone (unique contribution), and partly by the join contribution of other regressors, exerted through regressor correlation. In order to identify the contribution of each regressor and disentangle the role of correlation, a commonality analysis is performed [4]. This is a statistical tool to determine the partitioning of variance into the contribution given by each regressor into a multivariate linear regression. The following table shows that the magnitude is affected by the part conjointly explained by the other explanatory variables in the model. The commonality coefficients report a common value that increases over time (from 0.202 in 1990 to 0.325 in 2010) and a unique value of 0.003 for 1990, that increases at 0.010 for the 2000 cross section and 0.025 for year 2010.

Table A.3: Gravity model results: comparison with alternative methods. ZIP and ZINB are 2nd step results for year 2000. Results are estimated considering only active links on migration, while zero trade flows are considered.

Variable / Model	baseline	Poisson	NegBin	ZIP	ZINB
**migration**	0.185***	0.237***	0.327***	0.227***	0.264***
**weighted distance**	-0.862***	-1.037***	-0.984***	-1.011***	-0.775***
**population(o)**	0.836***	1.262***	1.361***	1.215***	1.175***
**population(d)**	0.681***	1.078***	1.291***	1.046***	1.165***
**per capita GDP(o)**	0.486***	0.663***	1.009***	0.637***	0.830***
**per capita GDP(d)**	0.558***	0.723***	1.100***	0.712***	0.930***
**contiguity**	0.297***	0.332***	0.697***	0.328***	0.717***
**common language**	0.190***	0.306***	0.213**	0.314***	0.252***
**colony**	0.469***	0.083***	0.589***	0.106***	0.485***
**common currency**	-0.140	0.258***	-0.381*	0.271***	-0.269*
**rta**	0.183***	0.042***	0.243*	-0.573***	0.196**
**tta (o)**	0.444***	0.826***	0.378***	0.772***	0.303***
**tta (d)**	0.0025	-0.858***	0.150	-0.151***	-0.011
θ **(overdispersion)**	-	-	0.115***	-	0.273***
**N. observations**	8874	11234	11234	11234	11234

Table B.1: Commonality coefficients for the baseline model (OLS method), where **uni** indicates the *unique contribution* of the variable alone and **tot** indicates the *total contribution* of the variable, including its cross-correlation with other variables.

Variable / Decade	1990		2000		2010	
	**uni**	**tot**	**uni**	**tot**	**uni**	**tot**
**migration**	0.003	0.202	0.010	0.260	0.025	0.325
**weighted distance**	0.021	0.026	0.024	0.026	0.021	0.058
**population (o)**	0.072	0.074	0.118	0.129	0.102	0.125
**population (d)**	0.089	0.099	0.071	0.074	0.063	0.083
**per capita GDP (o)**	0.020	0.012	0.036	0.026	0.022	0.017
**per capita GDP (d)**	0.038	0.007	0.032	0.007	0.012	0.001
**contiguity**	0.001	0.021	0.001	0.029	0.001	0.049
**common language**	0.002	0.000	0.002	0.001	0.003	0.002
**colony**	0.006	0.019	0.003	0.022	0.001	0.021
**common currency**	0.000	0.000	0.000	0.002	0.000	0.008
**rta**	0.000	0.020	0.001	0.048	0.003	0.061
**tta (o)**	0.007	0.029	0.011	0.024	0.003	0.008
**tta (d)**	0.000	0.010	0.000	0.001	0.001	0.000

C Different types of commodities

Virtual water fluxes are associated with the trade of different commodities and are summed across all commodities to obtain the total VW fluxes associated to trade (see the **Data** section in the main text). Commodities can also be grouped into categories to evaluate the associated VW flows; categories here considered are: crops, luxury food, animal-based and non-edible products. The list of products and corresponding categories can be found in [5]. We analyzed, by means of the OLS gravity model in Equation (1) (main text) and the FE specification in Equation (1) in the SI, the effect of migration on the VW trade associated to each category, and we found that all categories are positively affected by migrants (see the coefficients in the following table). The effect of migration is higher for VW trade associated to crops and animal-based commodities, while migrants have less importance in explaining VW trade associated to non edible commodities. Model coefficients also show an increasing temporal trend, as found in the analyses of total VW trade.

Table C.1: Migration coefficients in the OLS and FE gravity models applied to the VW flows associated to different categories of commodities. Results are estimated considering only active links on both trade and migration.

**Categories / Decade**	**1990**	**2000**	**2010**
**VW in crops - OLS**	0.186***	0.210***	0.345***
**VW in animal-based products - OLS**	0.165***	0.140***	0.197***
**VW in luxury food - OLS**	0.045***	0.132***	0.216***
**VW in non-edible products - OLS**	-0.124***	-0.079***	0.059***
**VW in crops - FE**	0.240***	0.309***	0.344***
**VW in animal-based products - FE**	0.241***	0.247***	0.179***
**VW in luxury food - FE**	0.189***	0.285***	0.280**
**VW in non-edible products - FE**	0.103***	0.149***	0.175***

D Migration flows data

It may be argued that the effect of migrants moving in a certain period (migration flows) can be significant, in place of that of migration stocks. However, we suspect a temporal delay between the migrants settlement and trade initiation/intensification thus we expect the effect of stocks to be higher. To check for this hypothesis, we used two different sources of data. First, we constructed migration flows data for each pair of countries and each direction from stock data, by taking the differences between subsequent decades (M_{2000}-M_{1990} for the decade 1990-2000, and M_{2010}-M_{2000} for the decade 2000-2010); occasional negative values are set to 0. Then, we used a more accurate migration flow database, built by Abel & Sanders [6] from the same UN stock data, but accounting for births and deaths rates [7]. They estimated migration flows for the periods 1990-1995, 1995-2000, 2000-2005, 2005-2010, that we summed up to match the decades of our analysis. We re-ran the OLS and the FE models for the two decades (see the following tables), and we computed commonality coefficients on OLS method using both our differenced UN data and Abel-Sanders data (**migration_AS** in the tables). Results highlight that also migration flows significantly affect the VW fluxes, but to a smaller extent compared to migrant stocks.

Table D.1: Regression results for the decade 1990-2000, using flows of migration instead of migration stocks. i) OLS with our flows, ii) OLS with Abel-Sanders flows, iii) FE with our flows, iv) FE with Abel-Sanders flows. Results are estimated considering only active links on both trade and migration.

**Variable / Application**	**i**	**ii**	**iii**	**iv**
**migration**	0.064***	\	0.092***	\
**migration_AS**	\	0.104***	\	0.203***
**weighted distance**	-1.004***	-0.864***	-1.214***	-1.171***
**population (o)**	0.922***	0.919***	\	\
**population (d)**	0.812***	0.787***	\	\
**per capita GDP (o)**	0.525***	0.563***	\	\
**per capita GDP (d)**	0.618***	0.726***	\	\
**contiguity**	0.385***	0.465***	0.334**	0.279***
**common language**	0.298***	0.296***	0.220***	0.271***
**colony**	0.544***	0.506***	0.597***	0.477***
**common currency**	-0.097	0.005	-0.201**	-0.224**
**rta**	0.210***	0.113*	-0.043	-0.019***
**tta (o)**	0.430***	0.463***	1.766***	1.621***
**tta (d)**	-0.141**	-0.096*	-0.085	1.072
**N. observations**	8388	8835	8500	8973
**R^2-adjusted**	0.443	0.432	0.668	0.659

Table D.2: Regression results for the decade 2000-2010, using flows of migration instead of migration stocks. i) OLS with our flows, ii) OLS with Abel-Sanders flows, iii) FE with our flows, iv) FE with Abel-Sanders flows. Results are estimated considering only active links on both trade and migration.

Variable / Application	i	ii	iii	iv
**migration**	0.110***	\	0.089***	\
**migration_AS**	\	0.184***	\	0.180***
**weighted distance**	-0.119***	-0.845***	-1.366***	-1.321***
**population (o)**	0.956***	0.912***	\	\
**population (d)**	0.811***	0.780***	\	\
**per capita GDP (o)**	0.474***	0.507***	\	\
**per capita GDP (d)**	0.488***	0.538***	\	\
**contiguity**	0.503***	0.577***	0.391***	0.349***
**common language**	0.417***	0.404***	0.293***	0.303***
**colony**	0.451***	0.380***	0.530***	0.438***
**common currency**	0.176*	0.069	0.035	-0.065
**rta**	0.290***	0.296***	0.174***	0.099*
**tta (o)**	0.207***	0.328***	1.396***	1.016***
**tta (d)**	-0.285***	-0.108*	0.361	-0.263
**N. observations**	8679	8162	8724	8162
**R^2-adjusted**	0.454	0.446	0.695	0.678

Table D.3: Commonality coefficients for the baseline model (OLS method), using migration flows derived from our dataset and from the Abel-Sanders dataset in two decades. **uni** indicates the *unique contribution* of the variable alone and **tot** indicates the *total contribution* of the variable, including its cross-correlations with other variables.

Variable / Decade	1990-2000				2000-2010			
	our flows		AS flows		our flows		AS flows	
	**uni**	**tot**	**uni**	**tot**	**uni**	**tot**	**uni**	**tot**
**migration**	0.002	0.097	0.004	0.175	0.006	0.136	0.012	0.217
**weighted distance**	0.036	0.022	0.022	0.023	0.039	0.052	0.020	0.044
**population (o)**	0.177	0.122	0.134	0.086	0.166	0.124	0.121	0.101
**population (d)**	0.127	0.081	0.100	0.066	0.113	0.079	0.090	0.069
**per capita GDP (o)**	0.044	0.027	0.050	0.031	0.033	0.017	0.039	0.030
**per capita GDP (d)**	0.042	0.007	0.067	0.034	0.023	0.000	0.029	0.013
**contiguity**	0.002	0.026	0.002	0.025	0.003	0.043	0.004	0.043
**common language**	0.005	0.000	0.004	0.002	0.008	0.002	0.007	0.004
**colony**	0.004	0.019	0.003	0.021	0.002	0.016	0.001	0.017
**common currency**	0.000	0.002	0.000	0.011	0.000	0.008	0.000	0.013
**rta**	0.001	0.046	0.001	0.055	0.003	0.060	0.003	0.066
**tta (o)**	0.010	0.030	0.010	0.019	0.002	0.013	0.004	0.016
**tta (d)**	0.001	0.002	0.000	0.003	0.002	0.002	0.000	0.004

E Instrumental variable approach

We used the instrumental variable approach to overcome the issues of potential reverse causality of migration and VW trade. Several approaches have been proposed in the literature [8, 9, 10] to remove the effect of spurious correlations using an instrumental variable. We adopt the two-stage least square method where, at the first stage, a regression is set between the migration flows and the time-lagged migration flows. At the second step, we use the fitted values of the first step as an exogenous migration variable in the *instrumented* regression. Looking at the migration coefficients in the instrumented regression (following table) compared with the same (non instrumented) coefficients (in this file, Section 4), it is possible to see that results are robust to different data and method applications, proving that the role of reverse causality in the VW trade-migrants relations is marginal.

Table E.1: Regression results for 2000-2010, using two-stage least square instrumental variable approach with time lagged migration flow as instrument. i) OLS, our flows, ii) FE, our flows, iii) OLS, Abel-Sanders flows, iv) FE, Abel-Sanders Flows. Results are estimated considering only active links on both trade and migration.

Variable / Application	i	ii	iii	iv
**migration**	0.183***	0.151***	\	\
**migration_AS**	\	\	0.202***	0.284***
**weighted distance**	-1.120***	-1.363***	-0.870***	-1.271***
**population (o)**	0.955***	\	0.964***	\
**population (d)**	0.830***	\	0.788***	\
**per capita GDP (o)**	0.482***	\	0.470***	\
**per capita GDP (d)**	0.499***	\	0.598***	\
**contiguity**	0.478***	0.381***	0.579***	0.311***
**common language**	0.426***	0.301***	0.443***	0.272***
**colony**	0.453***	0.540***	0.387***	0.393***
**common currency**	0.156	-0.006	0.094	-0.066
**rta**	0.285***	0.177**	0.307***	0.101*
**tta (o)**	0.196***	1.359***	0.360***	1.190***
**tta (d)**	-0.243***	0.120	-0.172**	-0.237
**N. observations**	8323	8345	6748	6748
**R^2-adjusted**	0.451	0.697	0.445	0.694

Considering the lagged migration flow variable as instrument and the results for our migration dataset only, the diagnostic tests provide the following results. The first-stage F-test for instrument relevance shows coefficients equal to 5047.6 (OLS gravity model) and 3372.3 (FE gravity model), both with very small p-value (< 0.0005); the Wu-Hausman F-test for esogeneity shows coefficients equal to 27.5 (OLS) and 8.9 (FE), both having p-value < 0.0005.

F The role of refugees and asylum seekers

An asylum seeker is defined as someone who leaves its own country, often for political reasons or because of war, and who travels to another country hoping that the government will protect him and allow him to live there. A refugee is an asylum seeker who obtained the permission to live in the destination country. These migrants which usually leave countries undergoing a crisis are unlikely to be capable of increasing the VW trade due to the food demand. For this reason, we think that a refugee does not cause VW fluxes, thus we used refugees data as a placebo test.

To check this hypothesis we perform the OLS and the FE gravity models for the period 2005-2010, replacing total migration flows with refugees and asylum seekers flows and averaging population and per capita gdp over the period 2005-2010. We used data from UNHCR Population Statistics Database [11], which reports information on refugees flows for every year for the period 2000-2013, where asylum seekers are taken as the people applying for asylum during the year, and the number of total decisions is taken as a proxy for the number of refugees. Results (in the following table) show a smaller effect of both refugees and asylum seeker on VW trade, which turns negative in the FE specification. Therefore, it is possible to argue that VW flows changes are affected only to a limited extent by the flow of forced migrants.

Table F.1: Regression results for the effect of Refugees/asylum seekers on VW trade. 2005-2010. i) OLS, refugees, ii) FE, refugees, iii) OLS, asylum seekers, iv) FE, asylum seekers. Results are estimated considering only active links on both trade and migration.

**Variable / Application**	**i**	**ii**	**iii**	**iv**
**refugees**	-0.086*	0.093***	\	\
**asylum seekers**	\	\	-0.088***	0.093***
**weighted distance**	-2.726***	-1.653***	-2.530***	-2.730***
**population (o)**	1.157***	\	1.126***	\
**population (d)**	1.050***	\	1.016***	\
**per capita GDP (o)**	0.424***	\	0.467***	\
**per capita GDP (d)**	0.848***	\	0.838***	\
**contiguity**	1.756***	1.666***	1.837***	1.837***
**common language**	1.234***	0.789***	1.037***	1.037***
**colony**	0.843***	0.703***	1.011***	1.011***
**common currency**	0.014	1.048*	-0.015	-0.015
**rta**	0.482**	0.290*	0.639***	0.639***
**tta (o)**	0.645***	2.557***	0.618***	0.618***
**tta (d)**	-0.763**	-1.059	-0.654***	-0.654***
**N. observations**	2607	2607	4764	4764
**R^2-adjusted**	0.388	0.665	0.424	0.661

References

[1] Anderson JE, Van Wincoop E. Gravity with gravitas: a solution to the border puzzle. National bureau of economic research; 2001.

[2] Baier SL, Bergstrand JH. Bonus vetus OLS: A simple method for approximating international trade-cost effects using the gravity equation. Journal of International Economics. 2009;77(1):77–85.

[3] Harrigan J. Openness to Trade in Manufactures in the OECD. Journal of international economics. 1996;40(1):23–39.

[4] Newton R, Spurrell D. Examples of the use of elements for clarifying regression analyses. Applied Statistics. 1967;p. 165–172.

[5] Tamea S, Allamano P, Carr JA, Claps P, Laio F, Ridolfi L. Local and global perspectives on the virtual water trade. Hydrol Earth Syst Sci. 2013;17.

[6] Abel GJ, Sander N. International migration flows; 2014. Accessed on 29/05/2014. Available at http://www.global-migration.info.

[7] Abel GJ, Sander N. Quantifying global international migration flows. Science. 2014;343(6178):1520–1522.

[8] Bratti M, De Benedictis L, Santoni G. On the pro-trade effects of immigrants. Review of World Economics. 2014;150(3):557–594.

[9] Ortega F, Peri G. The effect of income and immigration policies on international migration. Migration Studies. 2013;1(1):47–74.

[10] Peri G, Requena-Silvente F. The trade creation effect of immigrants: evidence from the remarkable case of Spain. Canadian Journal of Economics/Revue canadienne d'économique. 2010;43(4):1433–1459.

[11] UNHCR. UNHCR Population Statistics Database; 2014. Accessed on 25/05/2014. Available at http://popstats.unhcr.org/en/persons_of_concern.
